# Heart–Lung Interactions in Gas Exchange: From Physiology to Pathophysiology

**DOI:** 10.1002/cph4.70149

**Published:** 2026-04-17

**Authors:** Giuseppe Miserocchi, Egidio Beretta

**Affiliations:** ^1^ School of Medicine and Surgery University of Milano‐Bicocca Monza Italy

**Keywords:** AHRF, capillary transit time, lung edema, lung fibrosis, mechanical ventilation, perfusion limitation, surfactant

## Abstract

This review outlines the physiological organization of heart–lung coupling that ensures efficient gas exchange. Subsequently, factors that compromise this efficiency are examined. The development of perturbation in lung fluid balance, originating from both capillary and alveolar sources, is discussed in detail, as it represents a frequently overlooked contributor to impaired gas exchange. Intrinsic mechanisms by which the lung resists edema formation are then presented. We provide a quantitative functional model based on physical principles to describe the diffusion–perfusion interaction in the air–blood barrier aiming to interpret several pathological conditions, including pulmonary fibrosis, lung resection, heart failure syndromes, and mechanically ventilated patients with acute hypoxemic respiratory failure (AHRF).

## Graphical abstract

This review outlines factors compromising the efficiency of alveolar gas exchange. Perturbation in lung fluid balance from capillary and alveolar sources is considered. Safety factors against edema formation are presented. A model is presented to describe gas diffusion limitation considering pulmonary fibrosis, lung resection, heart failure syndromes, and mechanical ventilation.

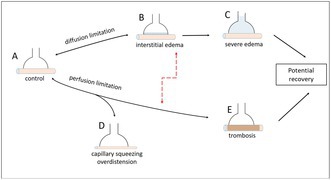

## 1 Morpho‐Functional Key Points

To accomplish gas exchange, the cardiovascular and respiratory systems operate within an integrated mechanical heart–lung framework. On the one hand, the heart acts as a tireless and powerful pump, capable of increasing its cardiac output by approximately fourfold, with a potential doubling of pulmonary arterial pressure. On the other hand, the lungs can accommodate these substantial changes in blood flow and inflow pressure while maintaining a relatively constant and low capillary blood pressure. This regulation is crucial to prevent the most dangerous consequence, namely fluid extravasation and the development of alveolar edema.

Under physiological conditions, heart–lung coupling ensures efficient gas exchange across the air–blood barrier (ABB), such that oxygen uptake precisely matches metabolic demand and carbon dioxide is removed accordingly. Gas exchange across the ABB can be defined as follows:

(1) $${\dot{M}}_{\mathrm{gas}}=D\cdot ∆P_{\mathrm{gas}}\cdot \frac{\text{surface of}\;\mathrm{ABB}}{\text{thickness of}\;\mathrm{ABB}}$$

where *D* is the diffusion–solubility coefficient and ${\Delta P}_{\mathrm{gas}}$ is the effective partial pressure gradient sustaining gas fluxes across the ABB. From a morphological standpoint, the lungs—despite their delicate yet mechanically robust architecture—appear to be oversized, allowing cardiac output to be distributed through approximately 2 × 10^9^ capillaries (Willführ et al. 2015) supplying about 3 × 10^8^ alveoli (Weibel 1984). This structural organization provides an estimated gas‐exchange surface area of ~100 m^2^ and maintains the thickness of the air–blood barrier at ~0.5 μm, including the endothelial and epithelial layers and the intervening interstitial compartment. Moreover, the high surface‐to‐thickness ratio enhances the efficiency of gas exchange. These exchanges involve a phase transition of respiratory gases: oxygen diffuses from the alveolar air into the blood, whereas carbon dioxide diffuses in the opposite direction for removal. Such phase transitions depend on specific biophysical properties of the gases, notably their diffusion–solubility coefficient in blood (the *D* term in Equation 1) and, for oxygen, its binding capacity to hemoglobin.

**FIGURE 1:**
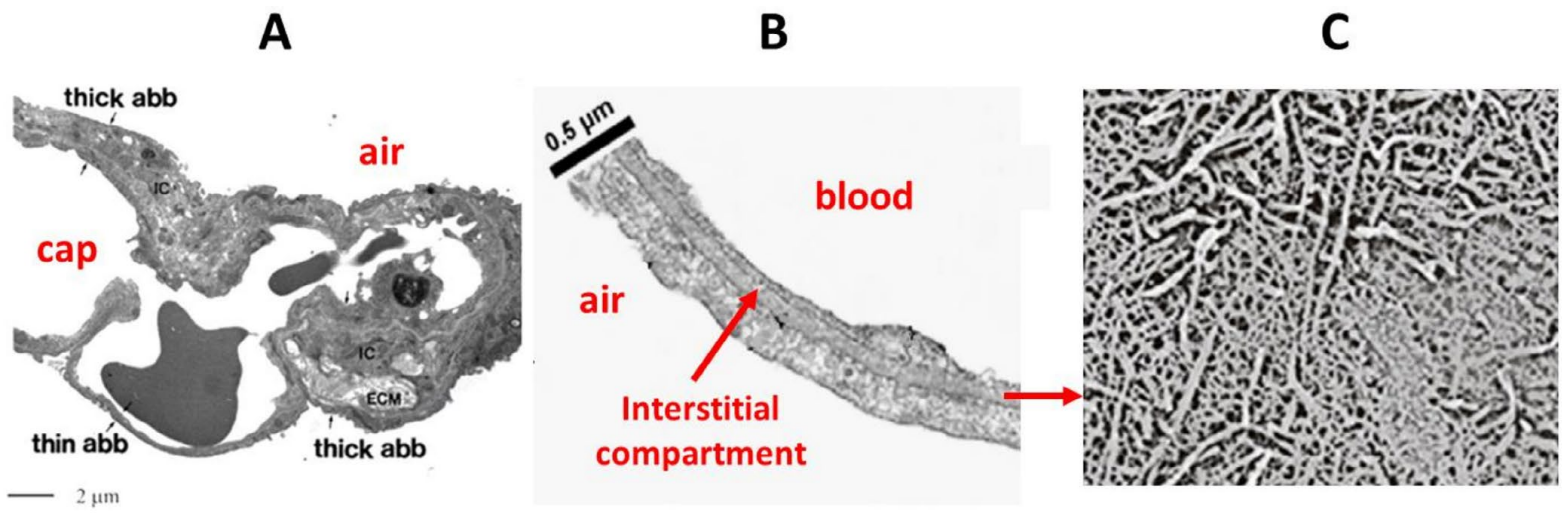
(A) Microphotograph of the air–blood barrier (ABB) (from Conforti et al. 2002). (B) Micrograph of the thin portion of ABB (from Conforti et al. 2002). (C) Macromolecular structure of the interstitial compartment. From Miserocchi (2023a).

Figure 1A shows a microphotograph of the ABB, whose extreme thinness (~0.5 μm) is highlighted in Figure 1B. The interstitial compartment (Figure 1C) should also be considered; it consists of a complex macromolecular network comprising (1) collagen and elastic fibers, which confer the mechanical properties of the lung, and (2) a large family of proteoglycans (PGs) that occupy the spaces between these fibers, providing compactness and rigidity to the overall molecular assembly. Proteoglycans also impart very low permeability to water fluxes.

## 2 Control of Lung Water Balance

The thinness of the ABB depends on the maintenance of a minimal amount of extravascular lung water. This review emphasizes the physiological role of the interstitial compartment in controlling the extravascular water content. Although this feature is relevant to all organs, it is particularly critical in the lung and the brain, where tissue water content must be strictly maintained at a minimum.

Water exchange between any two compartments is described by the so‐called Starling equation (Starling 1896), revised by Kedem and Katchalsky on thermodynamic basis (Kedem and Katchalsky 1958). In the context of capillary blood and the interstitial compartment (denoted by the subscripts *c* and *i*, respectively), Equation (2) defines the transendothelial water flux ($J_{v}$) as a function of the hydraulic pressure difference ($P_{c}$ − $P_{i}$) and the colloidosmotic pressure difference ($\Pi_{c}$ − $\Pi_{i}$):

(2) $$J_{v}=L_{p}\cdot A\cdot \left[\left(P_{c}-P_{i}\right)-\sigma \cdot \left(\Pi_{c}-\Pi_{i}\right)\right]$$

The proportionality factor $L_{p}$·*A* = $K_{f}$ represents the hydraulic filtration coefficient, defined as the product of the hydraulic permeability of the endothelium ($L_{p}$) and the surface area (*A*) available for fluid exchange. The coefficient *σ*, termed the reflection coefficient, was introduced by Kedem and Katchalsky (1958) to describe the permeability of biological membranes to nonelectrolytes, particularly proteins. The parameter *σ* reflects membrane permselectivity and is determined by the ratio between protein size and membrane pore size. Its value ranges from 0 (no restriction to proteins flow ‐ across the capillary) to 1 (impermeable to proteins). Neglecting *σ* and considering only the balance between hydraulic and colloidosmotic pressure differences led to markedly incorrect interpretations of the pathophysiology of fluid exchange. Equation (2) can be applied to analyze transendothelial and transepithelial water exchanges.

**FIGURE 2:**
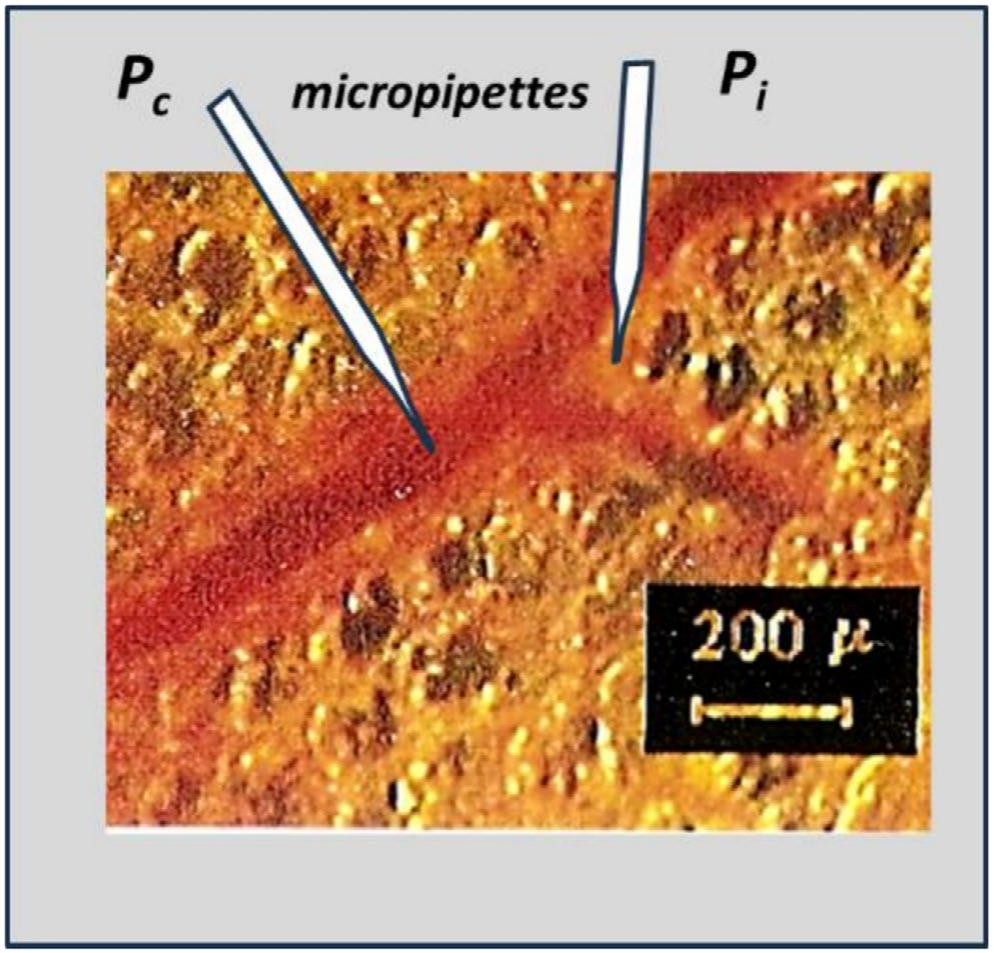
Microphotograph depicting the transpleural recording of microvascular and interstitial tissue pressures by micropuncture technique. Property of corresponding author.

From an experimental standpoint, accurate estimate of fluid balance across the capillary wall requires knowledge of *P*_*c*_, *P*_*i*_, $\Pi_{c}$ and $\Pi_{i}$, and *σ* values. The micropuncture technique was developed to measure *P*_*c*_ and *P*_*i*_ (Figure 2); however, it is limited to vessels with diameters greater than ~45 μm (Bhattacharya et al. 1989; Shepard et al. 1988). This technique remains so far the only minimally invasive approach allowing to measure *P*_*c*_ and *P*_*i*_ in intact closed chest in situ lungs. Using this approach, *P*_*c*_ was extrapolated to be approximately 10 cmH_2_O in lungs physiologically expanded within the chest (subatmospheric pleural pressure and atmospheric alveolar pressure) (Negrini et al. 1992).

The micropuncture technique was also employed to measure perimicrovascular interstitial pressure *P*_*i*_. Figure 2 shows a micrograph illustrating the transpleural recording of microvascular and interstitial tissue pressures. Under these conditions, *P*_*i*_ was found to be markedly subatmospheric, averaging −10 cmH_2_O (Miserocchi et al. 1990).

$\Pi_{c}$ was derived from protein plasma concentration, while $\Pi_{i}$ was obtained by the so‐called “wick technique” (Negrini, Candiani, et al. 2001; Negrini, Passi, et al. 2001) and estimated to be approximately ~ 30% of the plasma value.

**FIGURE 3:**
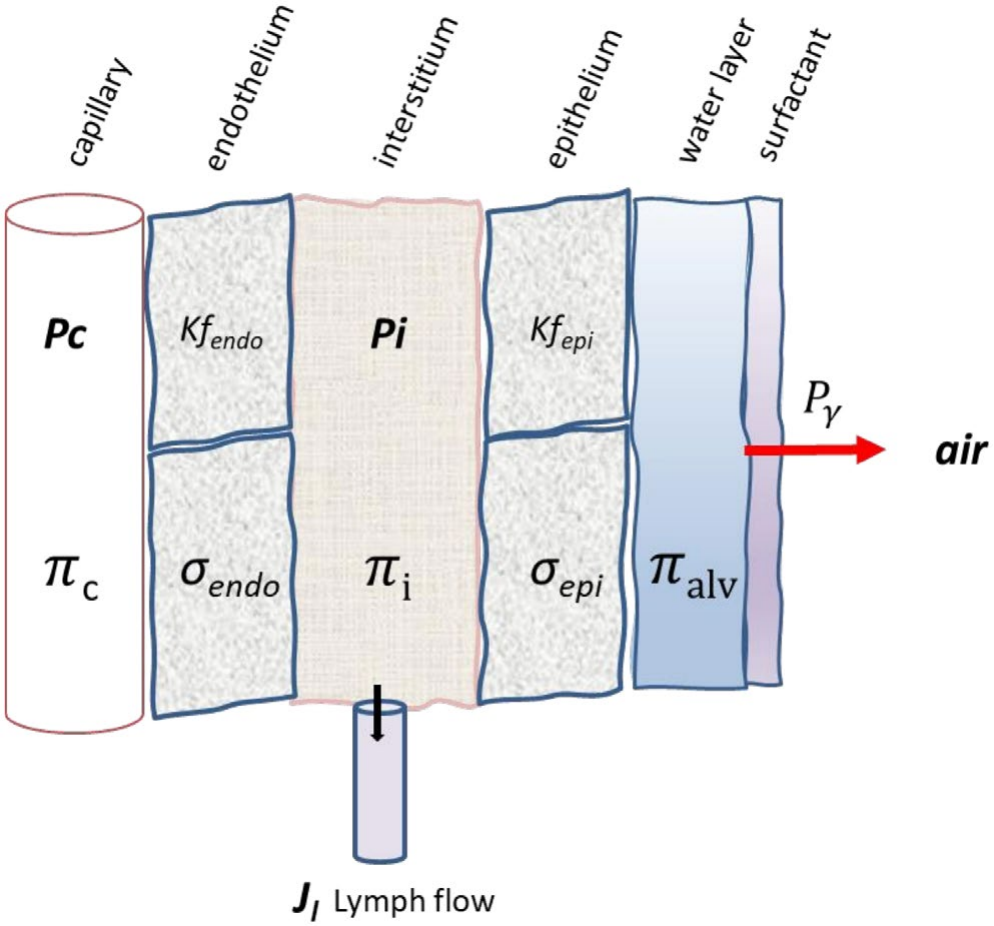
Lung fluid compartments of the air–blood barrier and parameters governing fluid exchanges. *P*_*γ*_ is the surfactant‐dependent surface tension (red arrow) exerted at the alveolar‐air interface; Π_alv_ is the oncotic pressure on the alveolar lining layer. See text for other symbols. Lymphatic drainage (*J*_*l*_) occurs from the thick portion of ABB where lymphatics are located (black arrow). Modified from Beretta et al. (2021).

Figure 3 schematically illustrates all compartments of the ABB to be considered when analyzing fluid exchanges according to Starling's law, from the capillary lumen to the alveolar surface.

At the endothelial level, the Starling pressure gradient favors fluid filtration into the interstitial space, being minimal in physiological conditions owing to the extremely low endothelial permeability (*L*_*p*_ in Equation 2) reflecting the dense meshwork of the macromolecular interstitial matrix. The structure of this matrix is subject to continuous and finely regulated dynamic remodeling. Capillary filtration is on the order of ~1 × 10^−4^ mL cm^−2^ day^−1^, an extraordinarily low value to maintain the thinness of the ABB at ~0.5 μm. By comparison, the diffusional fluxes of O_2_ and CO_2_ across the ABB are approximately 10,000‐fold greater (Miserocchi 2009).

On the alveolar surface a thin film of liquid is present whose thickness is ~0.15 μm; further, essentially no protein oncotic gradient has been detected across the pulmonary epithelium (Stephens et al. 1996). Given the negative value of *P*_*i*_, the resulting transepithelial pressure gradient favors reabsorption from the alveolar surface.

Lymphatic vessels are able to counterbalance the minimal interstitial water inflow (from capillaries and from alveoli) acting in fact as a “passive negative‐feedback control” tending to offset any increase in interstitial fluid volume (Miserocchi 2009). Interstitial fluid drainage occurs via initial lymphatics which offer no restriction to proteins (σ=0) and are able to generate a subatmospheric pressure (*P*_lymph_) lower than *P*_*i*_. Therefore, the pressure gradient generating lymphatic flow does not depend upon an oncotic pressure gradient being simply given by *P*_lymph_ − *P*_*i*_ (Miserocchi et al. 1989; Miserocchi 2009; Michel 1997). Noteworthy, lymphatics are sparse in the perialveolar region and therefore have a limited capacity for fluid drainage (Schraufnagel et al. 2003). On quantitative basis, lymphatics account for 100% of microvascular filtration in physiological conditions (Negrini et al. 1992; Miserocchi 2009).

### 2.1 The Mechanical and Vascular Safety Factors Against Lung Edema

These factors were identified by relying on experimental approaches inducing mild perturbations of lung fluid balance; this strategy allowed us to follow the temporal evolution of the pathophysiological mechanisms potentially leading to severe pulmonary edema. In contrast, models producing acute and severe lung injury proved to be inadequate to elucidate such mechanisms.

**FIGURE 4:**
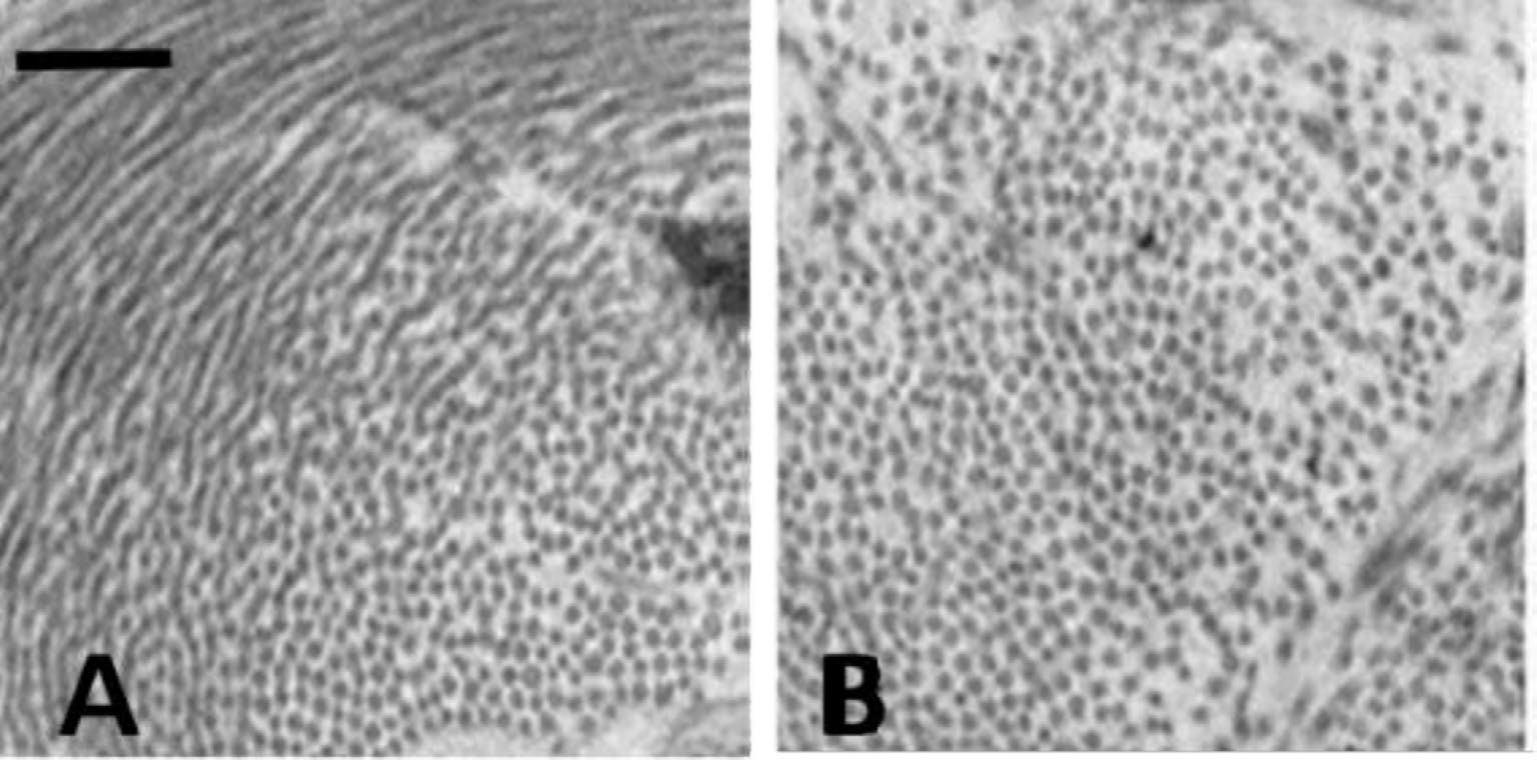
Cross sectional images of cluster of collagen fibers in the interstitial compartment of the septa in physiological conditions (A) and after development of interstitial edema (B). The white voids surrounding the fibrillary structure are occupied by hydrated proteoglycans. From Conforti et al. (2002).

Figure 4A shows clusters of cross‐sectioned collagen fibrils within the interstitial compartment of alveolar septa in a control lung; Figure 4B shows the increase in interfibrillar distance following the induction of mild interstitial edema causing hydration of the hyaluronan–versican complex to form a gel (data from Conforti et al. 2002).

**FIGURE 5:**
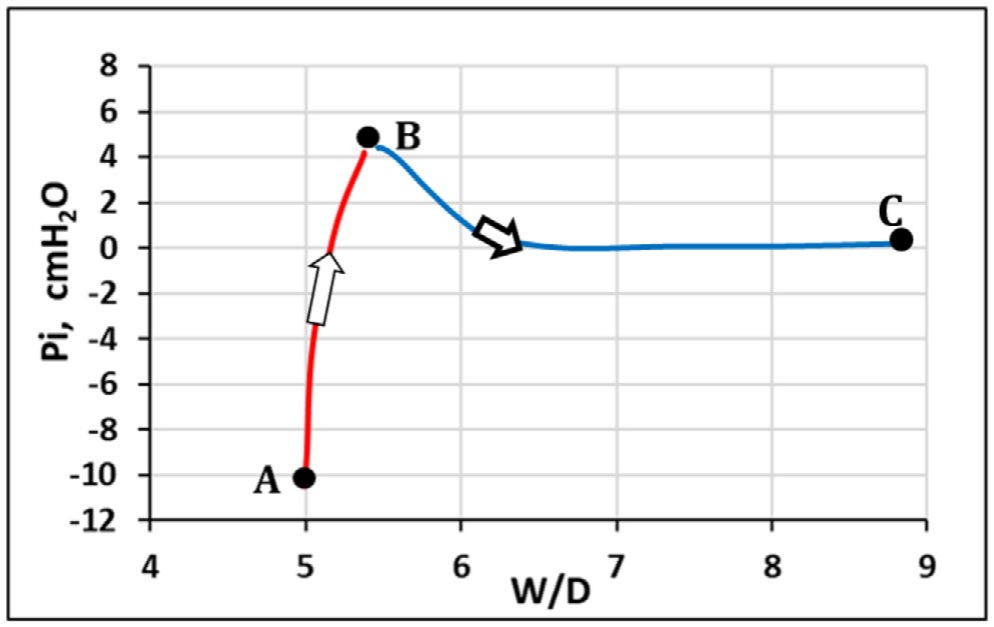
Mechanical response of the interstitial pressure to the increase of extravascular water: *P*_*i*_ increases from the physiological value of −10 cmH_2_O (point A) up to ~5 cmH_2_O (point B) for a 10% increase in *W*/*D*. Transition from point B to C (severe edema) is discussed later in the text. From Miserocchi (2023b).

As illustrated in Figure 5, the increase in steric hindrance of the gel raises $P_{i}$ from approximately −10 cmH_2_O to about +5 cmH_2_O (from point A to B, corresponding to panel A and B in Figure 4 respectively). The increase in extravascular lung water, as assessed by the wet‐to‐dry weight ratio (*W*/*D*), remains limited to ~10%. This disproportionate increase in $P_{i}$ relative to extravascular water content reflects the low compliance of the macromolecular interstitial matrix (Miserocchi et al. 1993, 2001; Negrini et al. 1996).

The elevation in $P_{i}$ acts as a “tissue safety factor” counteracting further filtration; moreover, since filtration is effectively halted, there is no requirement for increased lymphatic drainage (Miserocchi 2009).

The “vascular safety factor” relies on precapillary vasoconstriction of arterioles of approximately 80 μm diameter, documented in several edemagenic conditions (Parker et al. 1981; Audi et al. 1991; Clough et al. 2000; Negrini, Candiani, et al. 2001; Negrini, Passi, et al. 2001; Moudgil et al. 2005; Raj et al. 1990). From the standpoint of fluid balance, precapillary vasoconstriction prevents the increases in capillary hydraulic pressure, thereby attenuating microvascular filtration.

Notably, both the development of lung edema and the occurrence of precapillary vasoconstriction have been reported to be spatially and temporally erratic in the lung, suggesting a heterogeneous microvascular response to edemagenic stimuli (Rivolta et al. 2011; Hanaoka et al. 2000; Ngeow and Mitzner 1983).

**FIGURE 6:**
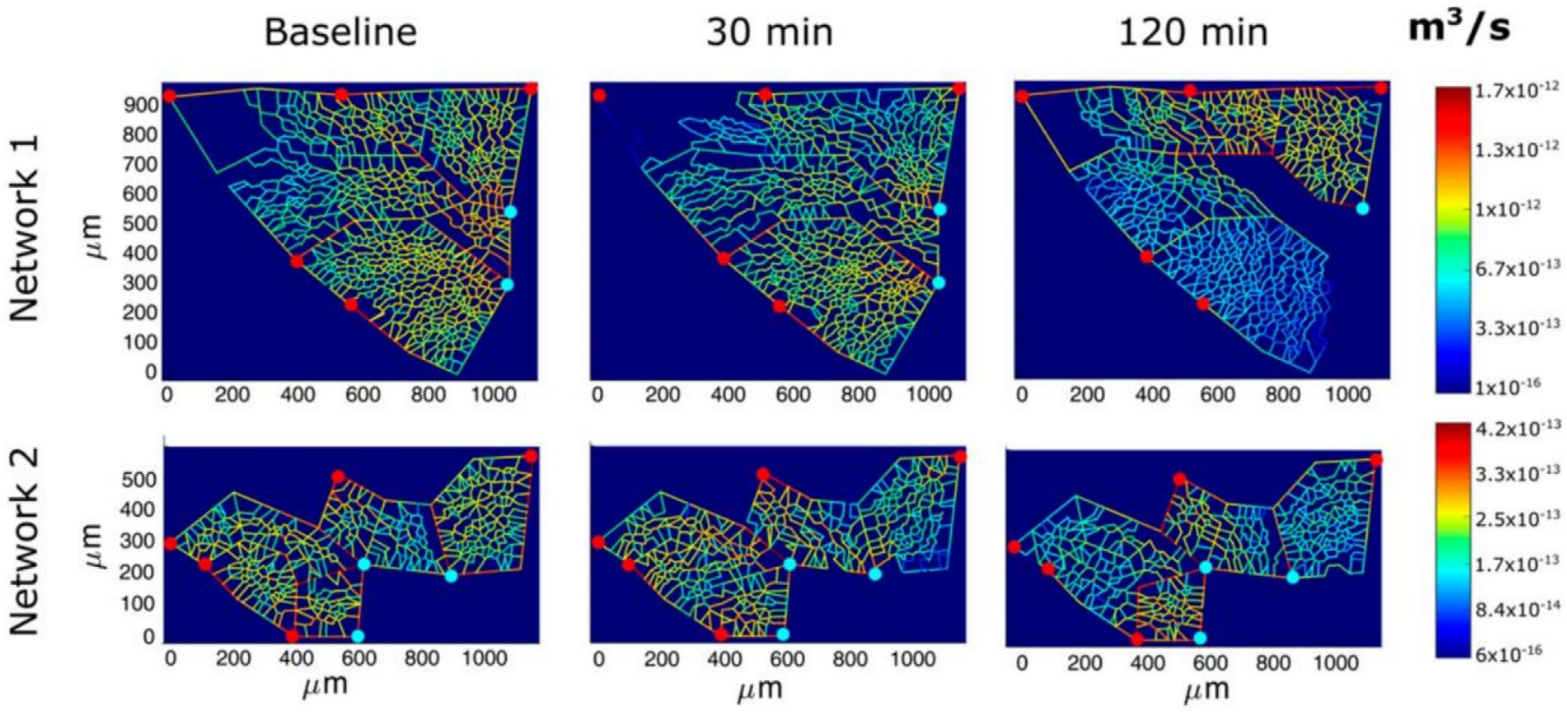
Perfusion analysis of alveolar‐capillary units in response to 12% hypoxia. Red and light blue dots denote arteriolar inlets and venular outlets, respectively. The color‐coded logarithmic scale ranges from yellow (vasodilation) to blue (vasoconstriction) over 30–120 min of hypoxic exposure. From Mazzuca et al. (2019).

A study by Mazzuca et al. (2019), using a hypoxia exposure model (Figure 6), demonstrated complete microvascular closure (blue on the color‐coded logarithmic scale) in regions where interstitial edema was developing, whereas vasodilation (yellow) was observed in non‐edematous regions. The authors proposed that the erratic spatial distribution of edema may correlate with local morphological features that favor fluid extravasation. The obvious fluid‐dynamic consequences of precapillary pulmonary vasoconstriction is the increase in pulmonary artery pressure (Dunham‐Snary et al. 2017; Groves et al. 1987; Moudgil et al. 2005).

### 2.2 Lung Edema From Capillary Origin

A critical question is how long the “mechanical safety factor” can withstand sustained edemagenic conditions? Chemical bonds of the PGs family are non‐covalent and therefore loose force on increasing intermolecular distance by the inverse square law; accordingly, binding forces fall off with the square of the distance between the interacting molecules. Accordingly, doubling of the intermolecular distance within the matrix mesh (e.g., from ~50 to ~100 nm, Figure 4B) would reduce the corresponding intermolecular forces to approximately one quarter of their original magnitude. Yet, a key determinant in the transition to severe edema is the progressive fragmentation of large PGs, triggered by: (1) reactive oxygen species generated during inflammation (bacterial, viral, or of sterile type, e.g., hypoxia or surgery); (2) mechanical stress (lung overdistension); and (3) activation of metalloproteinases (Miserocchi et al. 1999; Negrini et al. 1996, 1998; Passi et al. 1999). Loss of integrity of the interstitial mesh results in increased tissue compliance and enhanced microvascular permeability, reflected by a decrease in *σ* and an increase in $L_{p}$ (Equation 2). These factors, as depicted in Figure 5 (transition from point B to C), dissipate the increase in $P_{i}$, capillary filtration gradient is restored leading to the rapid development of severe edema.

An important clinical note is that, once the “tissue safety factor” is lost, the time constant for the development of severe edema is surprisingly short, on the order of 3–5 min (Mazzuca et al. 2016). In clinical conditions such as acute respiratory distress syndrome (ARDS), *W/D* ratios as high as 9 are commonly reported.

**FIGURE 7:**
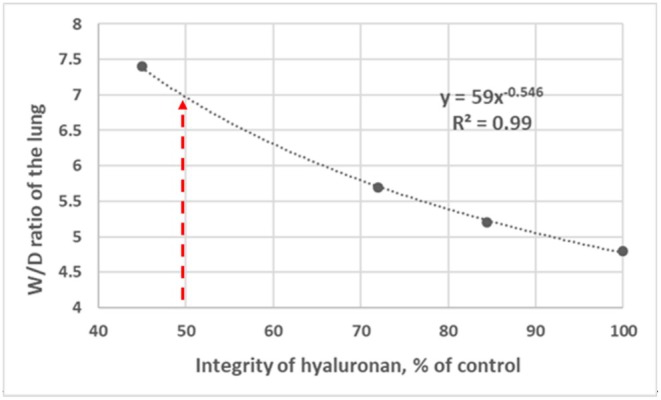
Relationship between the increase in *W/D* ratio of the lung and the loss of integrity of interstitial hyaluronan. Modified from Negrini et al. (1996).

Figure 7 illustrates that the extent of PGs fragmentation closely correlates with the increase in lung water, supporting the concept that disruption of the interstitial matrix is a critical determinant in the transition from controlled filtration to overt pulmonary edema (Negrini et al. 1996).

**FIGURE 8:**
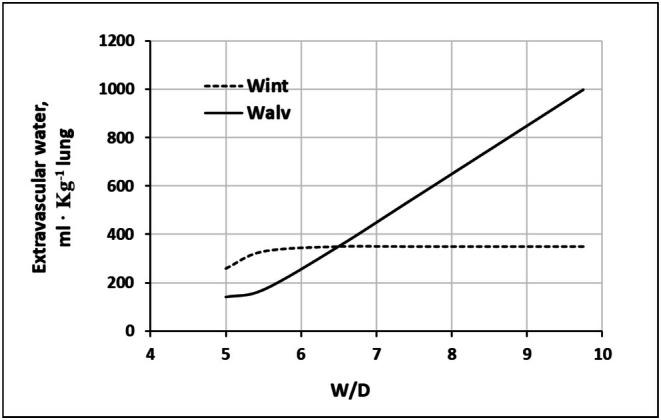
Distribution of extravascular lung water in the interstitial (*W*_int_) and alveolar (*W*_alv_) compartment on increasing *W/D* ratio. From Beretta et al. (2021).

A 50% reduction in native hyaluronan content is associated with a lung *W/D* of approximately 6.5 (Figure 7), which is considered a critical threshold beyond which severe pulmonary edema develops (Beretta et al. 2021). Beyond a *W/D* ratio of ~6.5 (Figure 8), water continues to accumulate within the alveolar compartment, suggesting saturation of the lung lymphatic drainage capacity (Beretta et al. 2021).

Lung overdistension beyond ~75% of total lung capacity represents an edemagenic factor, as it increases tensile stress along the alveolar septa (Miserocchi et al. 2008; Knudsen and Ochs 2018). Excessive stretch promotes disassembly of PGs within the interstitial matrix and along the endothelial glycocalyx, leading to increased permeability to water and solutes (Carlton et al. 1990; Vink and Duling 1996; Dehler et al. 2006; Miserocchi et al. 2001).

Pulmonary edema of varying severity has been reported in healthy humans performing prolonged exercise at ~75%–80% of $\dot{V}O_{2\max}$ for 2–3 h (Zavorsky 2007). In this context, the limited mechanical resistance of the pulmonary capillary wall to elevated $P_{c}$, a phenomenon termed “stress failure,” has been invoked in several experimental and clinical studies (West et al. 1991; West and Mathieu‐Costello 1992a, 1992b; Bachofen et al. 1993; Wu et al. 1995). In exercising humans at a workload of approximately 70 W, cardiac output doubles, pulmonary arterial pressure (PAP) increases from ~19 to ~30 mmHg, and pulmonary wedge pressure—commonly considered a surrogate of $P_{c}$—reaches ~20 mmHg (Langleben et al. 2019). Under these conditions, vascular resistance is estimated to decrease by ~23%, while capillary recruitment leads to an approximate doubling of the perfused capillary surface area.

### 2.3 Lung Edema From Alveolar Origin

Let's consider now lung edema from alveolar origin. In this case, the Starling transepithelial pressure gradient should consider the pressure generated by alveolar surface tension at the air interphase (*P*_*γ*_), defined as:

(3) $$P_{\gamma }=2\gamma /R$$

where *R* is the alveolar radius and *γ* is the surface tension. Under physiological conditions the surfactant layer assures *γ* approaching zero, so that *P*_*γ*_ ~ 0; the surfactant layer is kept stabilized by intercellular glycocalyx enriched in glycosaminoglycans and proteoglycans (Rizzo et al. 2022). Further, the Starling balance provides a pressure gradient of approximately 8 cmH_2_O to favor alveolar fluid absorption into the peri‐alveolar interstitial compartment. Mechanical stability for alveolar expansion is critically maintained by the rather subatmospheric *P*_*i*_ (~−10 cmH_2_O), assuring that the epithelial cells are kept well “glued” to the endothelial layer. The increase in *P*_*γ*_ due to surfactant deactivation (Gregory et al. 1991) represents the critical factor causing a shift of the Starling gradient from absorption to filtration leading to alveolar flooding.

**FIGURE 9:**
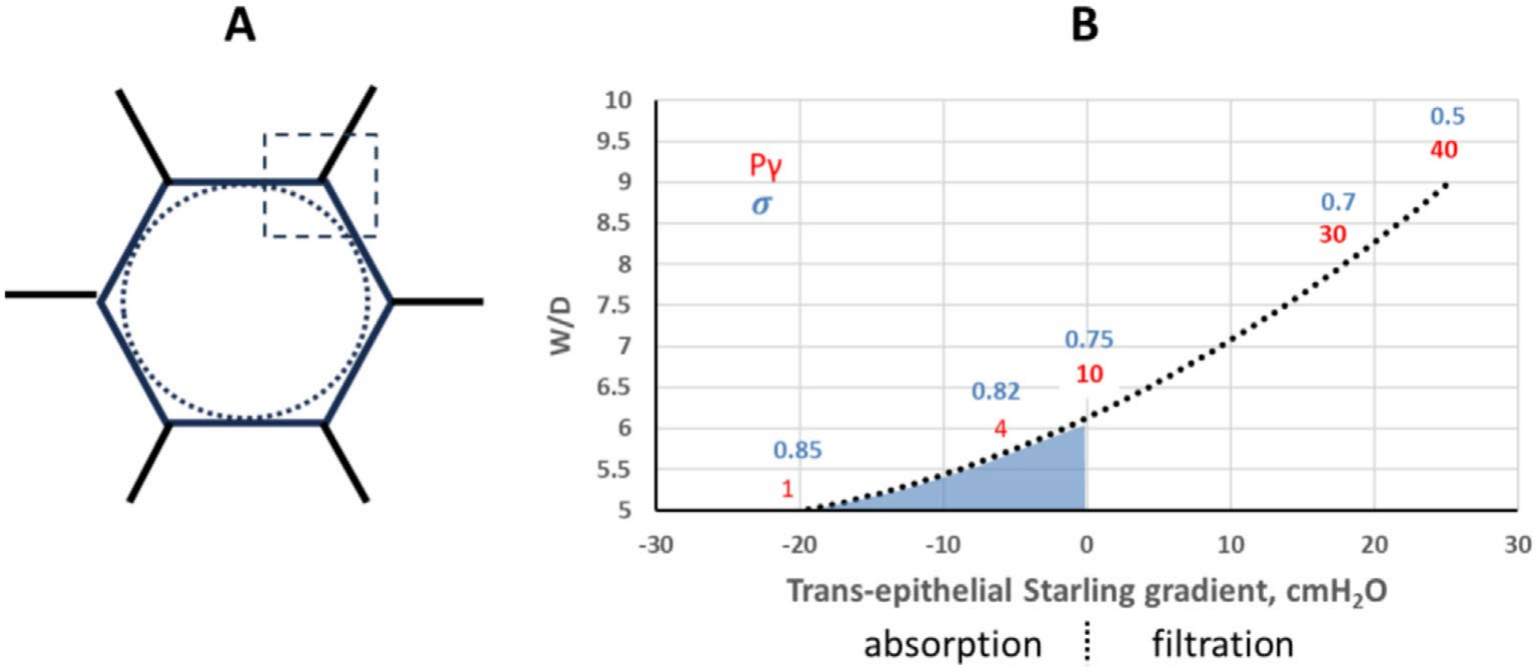
(A) The smaller radius of curvature is shown at corner site of an alveolus. (B) Modeling of the transepithelial gradient by progressively increasing *P*_*γ*_ and decreasing σ (increase in permeability). Negative values of the abscissa indicate gradient for alveolar fluid reabsorption toward interstitial space, positive values indicate gradient to cause alveolar edema. Blue area corresponds to the physiological range of variance. Redrawn from Nieman et al. (2025).

As originally pointed out by Guyton et al. (1976), the hexagonal geometry of alveoli is associated with a smaller radius of curvature at corner regions (Figure 9A), where $P_{\gamma }$ is expected to be higher. These sites may therefore be especially vulnerable to edema formation when surfactant integrity is compromised, as an increase in $P_{\gamma }$ would reduce or reverse the Starling transepithelial pressure gradient normally favoring fluid reabsorption.

Figure 9B shows how the increase in $P_{\gamma }$ and decrease in *σ* (increase in epithelial permeability) impact on the transepithelial Starling gradient to shift from absorption to filtration (negative and positive values respectively) to cause alveolar edema (increase in *W/D*). Under physiological conditions (*W/D* ≈ 5, $P_{\gamma }$ = 1, *σ* = 0.85) the transepithelial gradient favors alveolar fluid absorption. For an increase in $P_{\gamma }$ up to ~10 cmH_2_O and a decrease in *σ* dawn to ~0.75, the *W/D* approaches 6.5, the critical threshold nullifying the absorption gradient. Further increase in $P_{\gamma }$ and decrease in *σ* are strong factors leading to transepithelial filtration and alveolar flooding. The relationship shown in Figure 9B suggests that rise in $P_{\gamma }$ (up to ~40‐fold) is the dominant factor driving the increase in the filtration gradient, compared with the relatively modest (~50%) reduction in *σ*. Alveolar flooding develops via a self‐accelerating process (Hamlington et al. 2018).

The key issue to be considered is how an increase in *P*_*γ*_ retrogradely might affect the transcapillary pressure gradient. Indeed, an enhanced tendency for alveolar collapse caused by elevated *P*_*γ*_ should be associated with a corresponding negative shift in *P*_*i*_ that, in turn, would cause a transcapillary filtration gradient. These considerations support the hypothesis that surfactant deactivation may represent the early, largely hidden event triggering the development of alveolar edema (Buchholz et al. 2025). Mechanical ventilation contributes to surfactant degradation (Krischer et al. 2021). Further, alterations in surfactant composition and structure have been reported following high‐tidal volume mechanical ventilation (Maruscak et al. 2008). In contrast to edema originating at the capillary level, no effective safety factors appear to be available to counteract edema arising from the alveolar side of the ABB. Given the short time constant of developing severe edema, it appears crucial to have a diagnostic tool capable of detecting the early phase of development of edema.

### 2.4 Clearance of Alveolar Fluid

Under healthy conditions, the alveolar epithelium is almost totally impermeable to proteins (Gorin and Stewart 1979). In the presence of pulmonary edema, alveolar fluid reabsorption is performed by epithelial cells via active sodium‐dependent transport (Matthay 2014). This reabsorptive process is successful once the reparative process of interstitial matrix reorganization has occurred (Matthay and Wiener‐Kronish 1990), thus limiting/blocking further filtration. Indeed, alveolar fluid reabsorption is rapid in hydrostatic edema (implying modest increase in microvascular permeability) (Verghese et al. 1999), while in acute lung injury, alveolar fluid clearance is impaired by reduced Na^+^ absorption (Ware and Matthay 2001).

### 2.5 Early Signaling of Lung Water Perturbation

**FIGURE 10:**
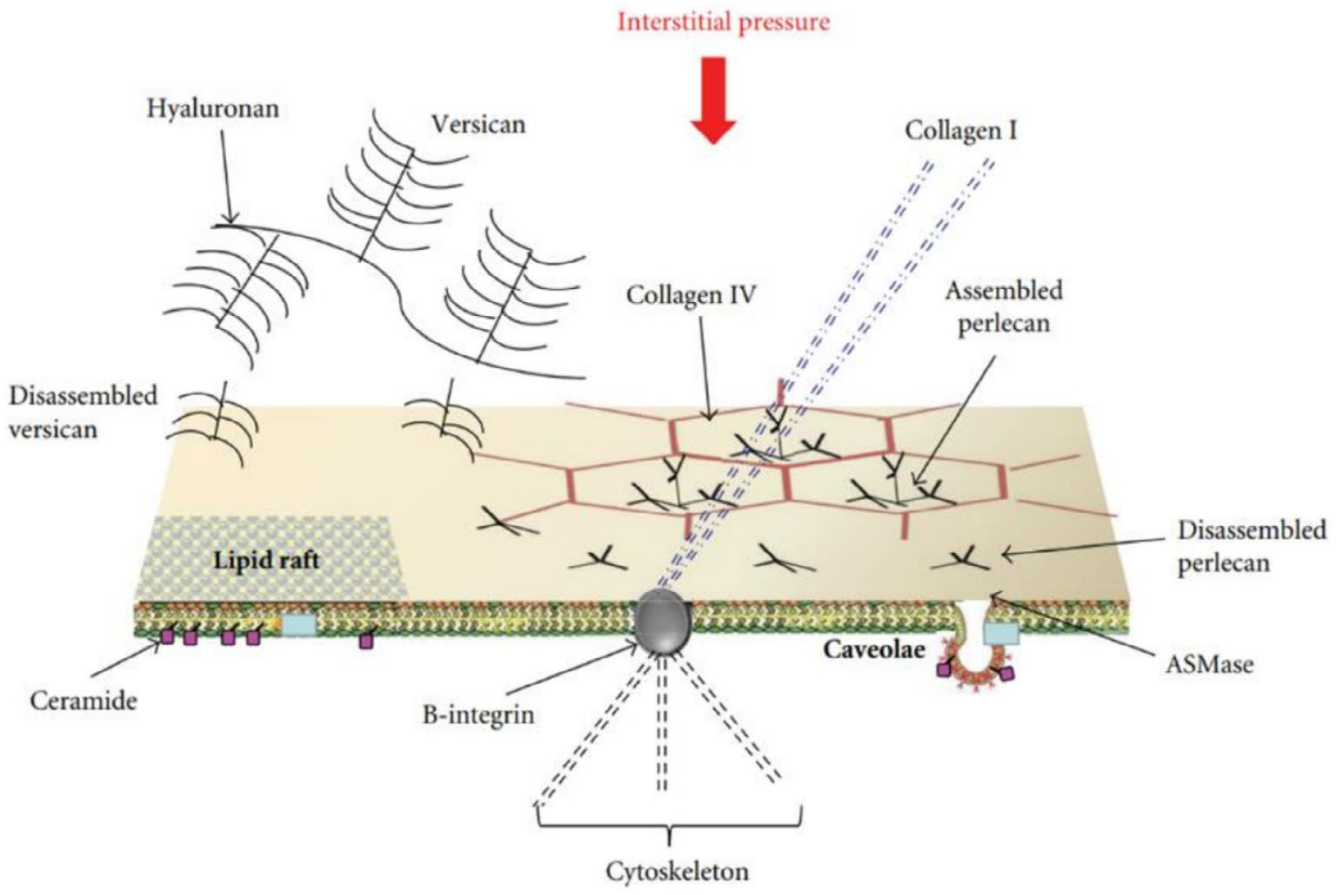
Model of lung cellular response triggered by increase in *P*_*i*_ in the early phase of developing lung edema (~10% increase of *W/D*). From Palestini et al. (2011).

Based on the marked increase in $P_{i}$ observed during the development of interstitial edema, it was hypothesized that this mechanical event may activate specific signaling pathways, as schematically illustrated in Figure 10. As suggested, increased $P_{i}$ in the early phase of interstitial edema development may act as a stimulus for mechanotransduction signaling in lung cells (Palestini et al. 2011).

Lipid rafts and caveolae at the plasma membrane are proposed to function as mechanosensors, responding to changes in the forces exerted by matrix proteoglycans (Florian et al. 2003). In this context, an increased expression of caveolae may facilitate interstitial‐to‐capillary water reabsorption, whereas downregulation (silencing) of aquaporin‐1 (AQP‐1) could limit transvascular water fluxes. Together, these coordinated cellular responses may represent an early adaptive mechanism aimed at counteracting fluid accumulation during the initial stages of pulmonary edema (Botto et al. 2006).

Further, activation of mechanosensitive ion channels is involved in the inflammatory response following endothelial barrier disruption due to an increase in pulmonary microvascular pressure, as on hypoxia exposure (Friedrich et al. 2019).

## 3 The Lung at Birth

At birth, the lungs are abruptly exposed to a marked increase in cardiac output; however, the ABB is not yet fully mature for gas exchange, as the alveoli are initially filled with lung‐derived liquid. The central question, therefore, is how this condition rapidly transitions to one that permits effective gas exchange at birth.

**FIGURE 11:**
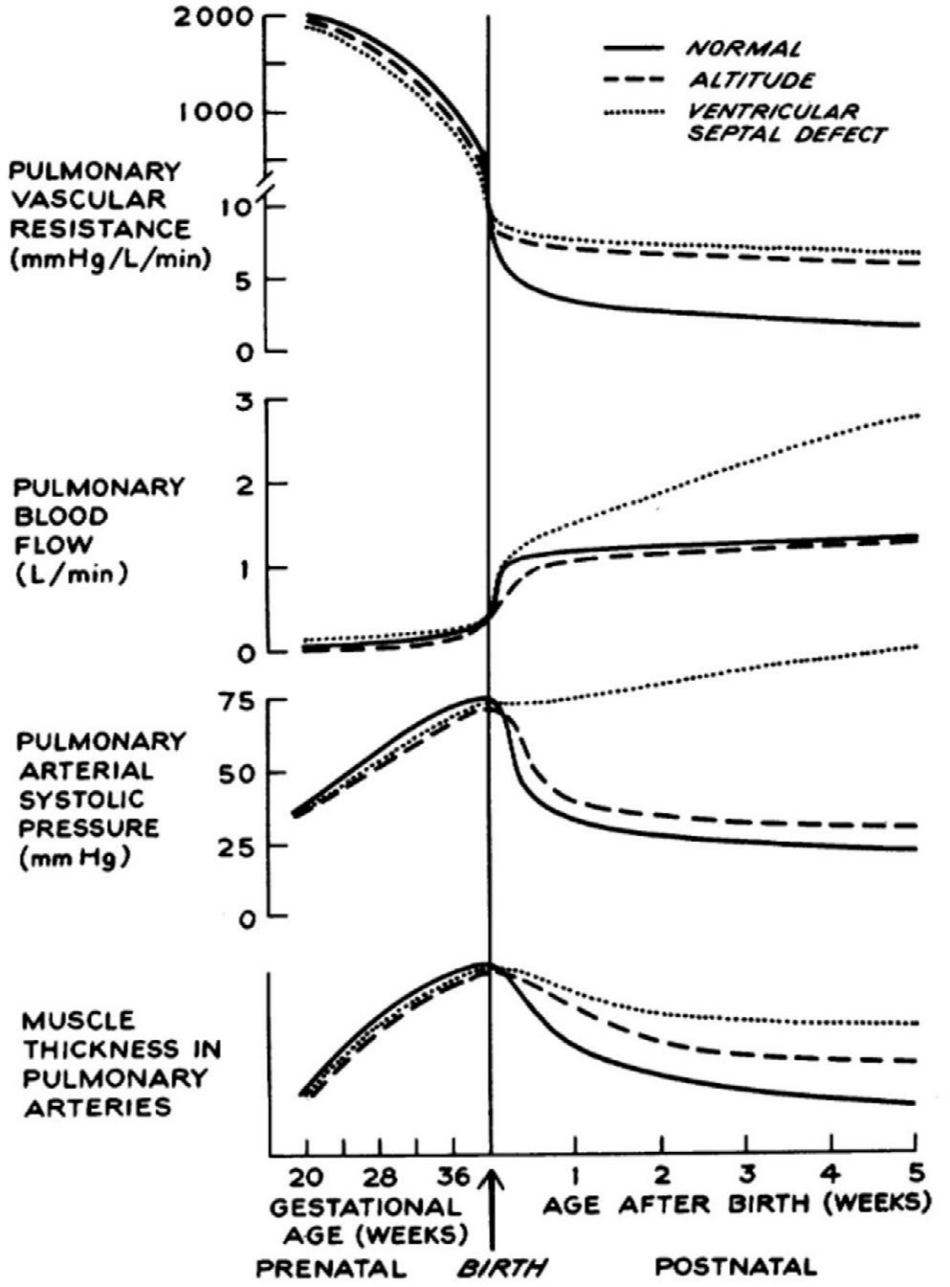
Time course of fetal and postnatal changes in pulmonary vascular resistance, pulmonary blood flow, pulmonary arterial systolic pressure, and thickness of smooth muscle in the medial layer of pulmonary arterioles. From Rudolph (1970).

In infants at approximately 1 h of life, mean pulmonary arterial pressure approaches—and may even exceed—systemic arterial pressure, reaching values of ~50 mmHg (Emmanouilides et al. 1964). This elevation reflects a substantial right ventricular afterload reflecting a complex equilibrium: on one side, the pulmonary capillary network ought to be fully distended and perfused; on the other, significant precapillary vasoconstriction is present as microvascular filtration ought to be avoided so as not to interfere with alveolar fluid drainage. Bidirectional shunting through the ductus arteriosus (left‐to‐right and right‐to‐left) persists during this early postnatal period. As shown in Figure 11, pulmonary arterial pressure then progressively declines, reaching normal levels by approximately 3 weeks of life (Rudolph 1970).

Precapillary vasoconstriction contributes to maintaining *P*_*c*_ at an estimated value of ~11 cmH_2_O (Bland and McMillan 1977). In term newborn rabbits, $P_{i}$, measured by micropuncture at birth, is approximately 0 cmH_2_O and increases to about 7 cmH_2_O within the first 2 h of life (Raj 1987; Miserocchi et al. 1994), suggesting a low compliance of the mature extracellular matrix. This early postnatal rise in $P_{i}$ may result from active alveolar fluid reabsorption (Jain and Eaton 2006), thereby generating a pressure gradient that promotes fluid drainage from the interstitial compartment into the pulmonary capillaries (Miserocchi et al. 1994). The majority of alveolar fluid clearance occurs via capillary absorption rather than through the lymphatic system (Bland et al. 1982). Over time, $P_{i}$ becomes progressively subatmospheric, in parallel with a reduction in the lung *W/D* ratio, indicating that most lung water clearance takes place within the first postnatal hours. By 16 days of life, $P_{i}$ approaches the physiological adult value of ~−10 cmH_2_O (Miserocchi et al. 1994).

In contrast, in non‐ventilated lung regions of premature rabbits, $P_{i}$ does not increase and remains close to 0 cmH_2_O (Raj 1987; Miserocchi et al. 1995), likely reflecting both the absence of active alveolar fluid absorption and/or a higher compliance of the macromolecular interstitial matrix. Surfactant deficiency would further impair alveolar fluid clearance and favor alveolar flooding.

We conclude this section by citing a recent perspective emphasizing that insufficient integration of cardiopulmonary physiology has limited progress in understanding heart–lung interactions during the neonatal and perinatal period, underscoring the need for renewed emphasis on physiological approaches in both training and clinical practice (McNamara et al. 2024).

## 4 Modeling Gas Exchange

Gas exchange depends on an effective interaction between ventilation and perfusion at the alveolar level. A widely used functional index to assess the efficiency of oxygen uptake is the ventilation/perfusion ratio (*V̇A/Q̇*) (Wagner 2008; Hopkins 2020; Hopkins et al. 2025). *V̇A/Q̇* ranges from 0, corresponding to perfused but non‐ventilated alveoli (shunt), to infinity, corresponding to ventilated but non‐perfused alveoli (dead space). The optimal condition is *V̇A/Q̇* = 1, indicating that alveoli are equally ventilated and perfused. This technique provides important insight to demonstrate ventilation/perfusion uncoupling in pathological condition.

A more quantitative description of gas diffusion and transport in blood was proposed by Piiper and Scheid (1981) based on the mass conservation principle, whereby the amount of oxygen diffusing across the ABB equals the amount transported by the blood. This model was originally developed to describe oxygen uptake along the length of the pulmonary capillary. More recently, the same model has been reformulated to express oxygen uptake and transport as a function of the time spent by the blood along the pulmonary capillary as presented below.

Based on the mass conservation principle one has:

(4) $$\dot{M}\left(t\right)=\dot{Q}\cdot \mathrm{dC}\left(t\right)$$

where $d\dot{M}\left(t\right)$ is the rate of oxygen diffusion as a function of time, which equals oxygen transport in blood, given by the product of cardiac output ($\dot{Q}$) by the increase in blood oxygen concentration (dC). Integration of this equation yields an exponential rise toward an equilibrium value ($L_{\mathrm{eq}}$) reached as blood exits the capillary. This equilibrium can be expressed in two equivalent ways. First, in terms of the variables governing diffusion and transport:

(5) $$L_{\mathrm{eq}}=e^{-{\mathrm{DO}}_{2}/\left(\beta \dot{Q}\right)}$$

where ${\mathrm{DO}}_{2}$ is the effective oxygen diffusive conductance of the lung, β is the oxygen‐binding capacity of hemoglobin, and $\dot{Q}$ is cardiac output.

Alternatively, equilibrium can be expressed as a function of capillary transit time (*Tt*) and the time constant (*τ*) of the equilibration process:

(6) $$L_{\mathrm{eq}}=e^{-\mathrm{Tt}/\tau }$$

The parameter $L_{\mathrm{eq}}$ varies from 0, indicating perfect equilibration, to 1, indicating complete absence of equilibration.

The time constant *τ* is defined as (Beretta et al. 2019; Miserocchi et al. 2022):

(7) $$\tau =\frac{\beta V_{c}}{{\mathrm{DO}}_{2}}$$

where $V_{c}$ is the volume of blood within the pulmonary capillary network participating in gas exchange.

Solutions of Equations ((5), (6), (7)) require knowledge of all the variables that can be experimentally measured.

Under resting physiological condition, $\frac{{\mathrm{DO}}_{2}}{\beta \dot{Q}}$ is approximately 10, indicating that lung diffusive capacity is largely redundant relative to blood oxygen transport capacity. The ratio $\frac{{\mathrm{DO}}_{2}}{\beta \dot{Q}}$ provides a meaningful index of heart–lung interaction as its decrease entails a lack of alveolar‐capillary equilibration.

**FIGURE 12:**
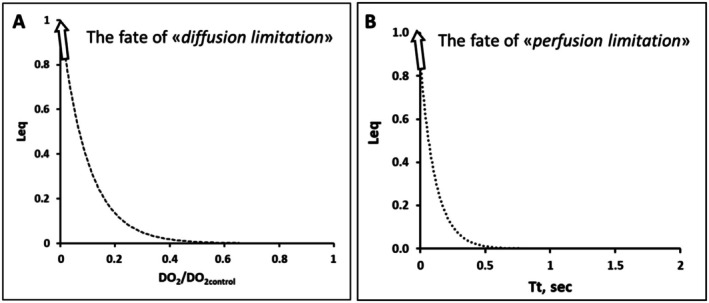
(A) Exponential increase of *L*_eq_ up to 1 on decreasing DO_2_ relative to its physiological value (case of diffusion limitation). (B) Exponential increase of *L*_eq_ due to shortening of the capillary transit time *Tt*. From Miserocchi et al. (2024).

Loss of efficiency in gas equilibration can initially be attributed to a reduction in ${\mathrm{DO}}_{2}$ (the numerator of Equation 5), a condition termed diffusion limitation. Pulmonary edema markedly decreases ${\mathrm{DO}}_{2}$, and as shown in Figure 12A, a fivefold reduction leads to an exponential rise in $L_{\mathrm{eq}}$ (Miserocchi et al. 2024). The denominator of Equation (5), representing blood O_2_‐carrying capacity, also influences gas exchange. A decrease in *β*, as seen in anemia, initially elevates the $\frac{{\mathrm{DO}}_{2}}{\beta \dot{Q}}$ ratio; however, the compensatory increase in cardiac output ($\dot{Q}$) to meet peripheral oxygen demands predominates, ultimately lowering $\frac{{\mathrm{DO}}_{2}}{\beta \dot{Q}}$ (Miserocchi et al. 2022; Bartesaghi et al. 2014).

Blood capillary transit time is also playing an important role in gas equilibration as shown by the function presented in Figure 12B mathematically derived from Equation (6) and (7) based on data experimentally obtained in humans breathing spontaneously (Miserocchi et al. 2024). Note that about half of total time spent by blood in pulmonary capillary is required for O_2_ to diffuse through the alveolar and the red cell membranes. The data range for blood capillary transit time (Figure 12B) is in accordance with theoretical estimates based on microfluidic models for Reynolds number in the range of 0.005 (Zhuang et al. 1983). According to Equation (6), elevated $\dot{Q}$ values accelerate blood flow velocity, reducing capillary transit time. For *Tt* falling below ~0.5 s, the exponential increase in loss of O_2_ equilibration was defined as “perfusion limitation.” Shortened *Tt* may additionally reflect vasoconstrictive responses. The final value of $L_{\mathrm{eq}}$ may thus reflect the combined effects of diffusion and perfusion limitation (Figure 12A,B).

### 4.1 Interindividual Differences

Interindividual variability in gas exchange has been interpreted within the framework of diffusion–perfusion limitation model considering that $\frac{{\mathrm{DO}}_{2}}{\beta \dot{Q}}$ is a key functional parameter to describe the cardiopulmonary interaction for oxygen uptake (Miserocchi et al. 2022; Miserocchi and Beretta 2023).

**FIGURE 13:**
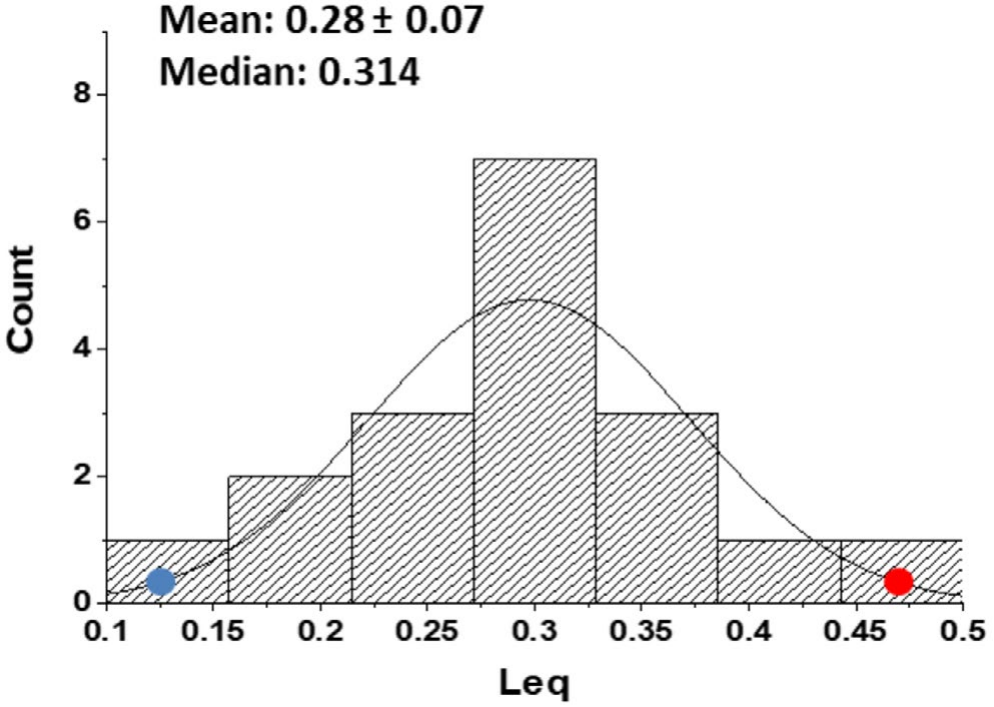
Interindividual variability of *L*_eq_ reflecting the functional value of the $\frac{{\mathrm{DO}}_{2}}{\beta \dot{Q}}$ ratio in response to a given metabolic requirement. The distribution of *L*_eq_ *i*s normal. From Miserocchi et al. (2022).

Figure 13 illustrates the interindividual distribution of $L_{\mathrm{eq}}$ under conditions requiring the functional change of the $\frac{{\mathrm{DO}}_{2}}{\beta \dot{Q}}$ ratio to meet a given metabolic demand. The distribution of $L_{\mathrm{eq}}$ is approximately normal, suggesting relative higher (blue point) or lower (red point) inborn efficiency of the heart–lung coupling for oxygen uptake facing the same metabolic requirement.

**FIGURE 14:**
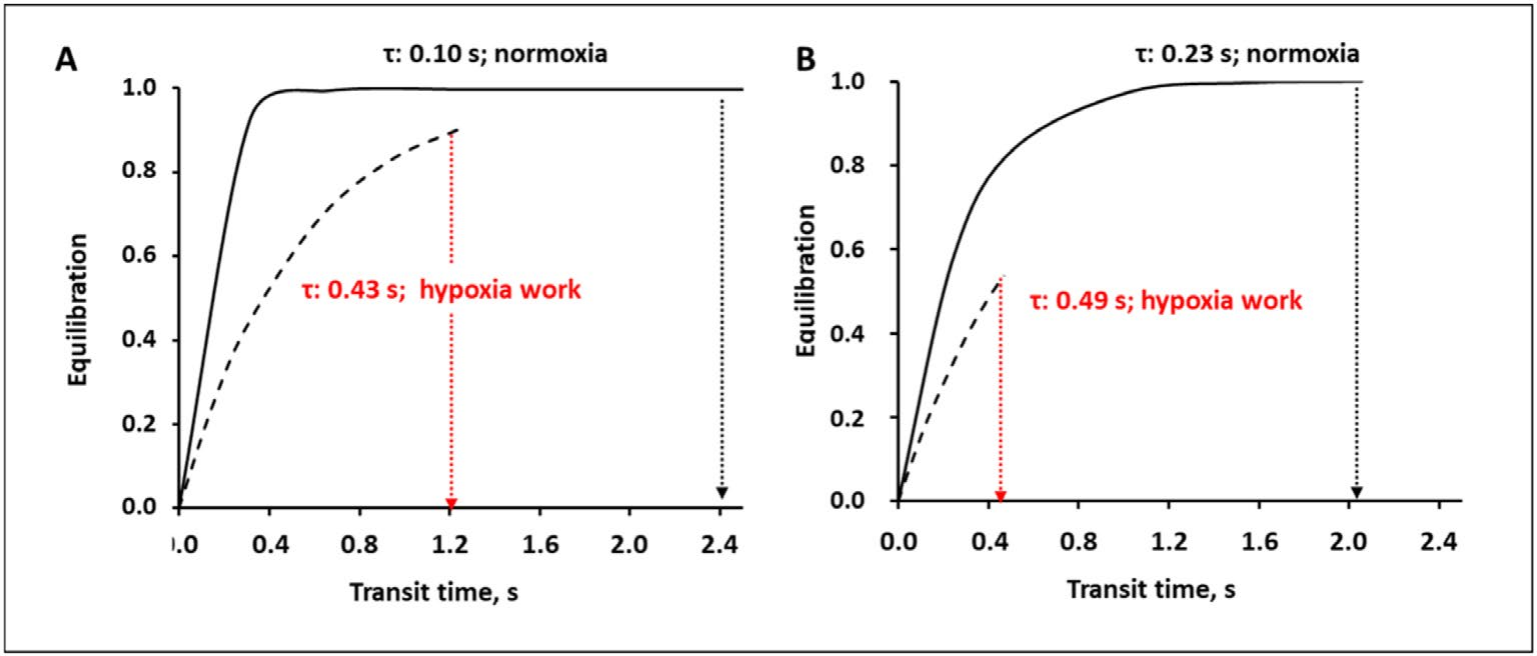
Interindividual differences in time course of the alveolo‐capillary equilibration in normoxia and in hypoxia. From Miserocchi et al. (2022).

Figure 14 illustrates the time course of alveolar O_2_ uptake in two subjects with differing intrinsic equilibration efficiency (blue and red dots in Figure 13, representing higher and lower efficiency, respectively). For clarity, the equilibration process is depicted as a rising curve, with the ordinate representing $1-L_{\mathrm{eq}}$, where a value of 1 denotes complete alveolo‐capillary equilibration. In Figure 14A, the high‐efficiency subject (blue dot) achieves full O_2_ equilibration under normoxic conditions (solid line) for *Tt* < 0.4 s and *τ* = 0.1 s. The low *τ* value (Equation 7) reflects a high alveolar diffusion capacitance (${\mathrm{DO}}_{2}$) relative to the capillary blood volume ($V_{c}$) contributing to O_2_ uptake. Under hypoxic work conditions (dashed line), equilibration is partially impaired due to an increase in *τ*, likely indicating a reduction in ${\mathrm{DO}}_{2}$. The concomitant decrease in *Tt* results from elevated cardiac output and increased blood velocity. Figure 14B depicts a subject with lower intrinsic O_2_ uptake efficiency (red dot), where complete equilibration under normoxia requires ~1.2 s. In this individual, the hypoxia‐induced increase in *τ* is more pronounced than in Figure 14A, reflecting a greater decrease in ${\mathrm{DO}}_{2}$ that is partially compensated by increased cardiac output. This compensation leads to a marked reduction in *Tt* (from ~2 to 0.4 s), representing the primary perfusion‐limiting factor.

It was hypothesized that subclinical disturbances in lung fluid balance may contribute to reduced ${\mathrm{DO}}_{2}$. This was investigated using the forced oscillation technique (FOT), which links subtle changes in lung fluid balance to measurable alterations in pulmonary mechanics (Dellacà et al. 2008). FOT analyses demonstrated that varying degrees of subclinical fluid imbalance limited O_2_ uptake efficiency by decreasing $\frac{{\mathrm{DO}}_{2}}{\beta \dot{Q}}$ while simultaneously increasing the time constant $\frac{\beta V_{c}}{{\mathrm{DO}}_{2}}$ (Bartesaghi et al. 2014).

Interindividual differences in alveolar O_2_ uptake efficiency were further examined in relation to alveolar‐capillary phenotype (Miserocchi et al. 2022). A morpho‐functional model suggested that relatively smaller alveoli (implying a higher alveolar density per unit lung volume) confer several functional advantages (Miserocchi et al. 2008, 2022; Miserocchi 2023b):

1. Higher $\frac{{\mathrm{DO}}_{2}}{\beta \dot{Q}}$ ratio and lower *τ*;
2. An anatomical design more resistant to edema, with relatively lower capillary blood volume compared to overall alveolar diffusion surface area;
3. Lower intrinsic microvascular permeability (lower *A* and possibly higher *σ* in Equation 2).

## 5 Pathophysiology of Gas Exchange

**FIGURE 15:**
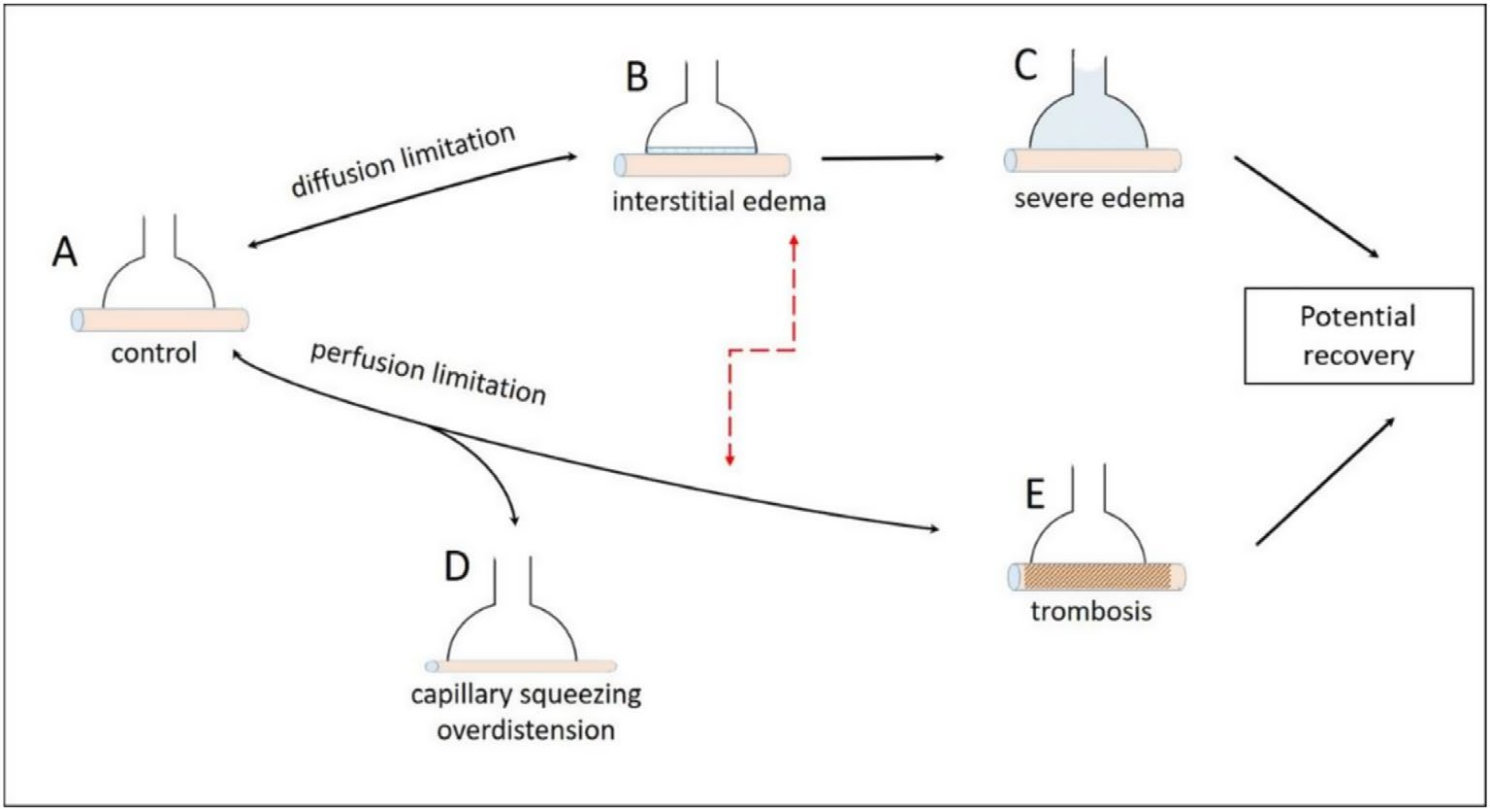
Schematic illustration of conditions that may adversely affect gas exchange during the development of pathological states. From Miserocchi et al. (2024).

Figure 15 shows a schematic illustration of conditions that may adversely affect gas exchange during the development of pathological states. From the physiological condition (A), *diffusion limitation* may develop as a consequence of interstitial edema (B), potentially progressing to severe edema with alveolar flooding (C). Under these conditions, a progressively increasing *shunt effect* develops. Panels A–E illustrate the case of *perfusion limitation*, resulting from vasoconstriction, capillary closure due to compression (e.g., increased alveolar pressure), or thrombosis. The progression from A to E represents an increasing contribution of *dead space*. Red dashed arrows indicate mixed mechanisms that may coexist in advanced lung pathology. Fibrosis represents the possible endpoint during the recovery or chronic phase. Concerning the potential increase in ABB thickness on recovery, a comment is due concerning the difference between arithmetic and harmonic mean thickness (Conforti et al. 2002). By nature, the thickness of the ABB is irregular, including the so‐called “thin” and “thick” portions. The arithmetic mean thickness reflects the mass of the tissue in the ABB, while the harmonic mean thickness is weighted toward the thinner portion, thus representing the resistance to gas diffusion (Weibel and Knight 1964). Interestingly, in experimental model of perturbation of lung fluid balance (Conforti et al. 2002), the arithmetic mean thickness increased, while the harmonic mean thickness remained low due to local interstitial fluid accumulation in the ABB, thus preserving the low thickness in the majority of the ABB. This represents an interesting functional adaptation to preserve gas diffusion properties.

### 5.1 Lung Fibrosis

Lung fibrosis is a connective tissue disease within the general family of systemic fibrosis due to excessive extracellular matrix deposition reflecting abnormal fibroblast activation. Over time, the progress of disease leads to vascular damage and endothelial dysfunction interfering with gas exchange both at pulmonary and systemic level (Alcolea et al. 2025).

Lung fibrosis has a strong negative impact on cardiopulmonary interaction, promoting the development of pulmonary hypertension (Munson 2010). On pathophysiological ground, pulmonary hypertension and precapillary vasoconstriction have been shown to be more pronounced in subjects who exhibit a greater perturbation of lung fluid balance on exposure to hypoxia, a strong edemagenic factor; these subjects display a higher‐than‐normal ratio of capillary density to alveolar surface area (Bartesaghi et al. 2014). Comparable morpho‐functional alterations have been reported in patients with pulmonary fibrosis; notably, with progression of fibrosis, the capillary density–to–alveolar surface area ratio decreases (Ebina et al. 2004).

Recent evidence from biopsies indicates that in the age range 2–18 years (Fan et al. 2015; Griese et al. 2024) within the frame of interstitial lung disease, fibrotic processes may develop in inflammatory conditions associated with increased microvascular permeability (hemorrhages and lung edema).

In parallel, potential proteomic biomarkers have been identified for interstitial lung disease and pulmonary hypertension in systemic sclerosis (Mismetti et al. 2023), offering new perspectives for prognosis and disease monitoring.

Based on these observations related to lung fibrosis, we propose as a working hypothesis that fibrotic tissue deposition may constitute a form of functional adaptation in individuals with intrinsically increased pulmonary capillary permeability and, consequently, a heightened susceptibility to pulmonary edema. Disease progression would then reflect the phenotypic trans‐differentiation of resident lung fibroblasts into contractile myofibroblasts, a process currently regarded as a key therapeutic target (Gan et al. 2022).

Given that pulmonary fibrosis inevitably leads to diffusion and perfusion limitations, a critical question is how its development can be diagnosed, particularly during the early stages, before functional impairment becomes clinically evident and irreversible. We propose two complementary strategies. From a functional perspective, disease monitoring should focus on: lung compliance and gas diffusion capacity. Lung compliance ought to be assessed by high‐frequency oscillatory techniques (FOT) (King et al. 2020). In parallel, gas diffusion capacity may be evaluated using the experimental–analytical framework described in Section 4, which provides a robust tool for the integrated assessment of cardiopulmonary dysfunction.

### 5.2 Lung Resection

The strategy for re‐expansion of the resected lung within the thoracic cavity has long been a matter of debate in chest surgery. With the availability of modern chest drainage systems, complete re‐expansion of the remaining lung has been advocated according to the so‐called “open lung strategy.” However, this approach has not fully explained the occurrence of severe postoperative complications such as pulmonary edema and hydrothorax.

Complete removal of gas from the pleural space represents a major cause of lung overdistension; importantly, the degree of overdistension increases with the volume of lung tissue resected. From a vascular standpoint, lung resection reduces the pulmonary capillary network, resulting in overperfusion of the remaining vasculature and an increase in capillary flow resistance. The fluid‐dynamic consequences include an elevation of capillary pressure and an increase in blood velocity within the alveolar‐capillary bed. Increased capillary pressure constitutes a potent edemagenic factor, whereas increased blood velocity may impair alveolar gas equilibration by shortening capillary transit time.

Lung overdistension represents the common pathophysiological mechanism underlying the main postoperative respiratory complications, namely persistent air leak, pulmonary edema, and hydrothorax (Miserocchi et al. 1991; Dreyfuss et al. 1988). On this basis, a mechanical analysis was developed to define appropriate levels of postoperative suction pressure, with the aim of exposing the resected lung to a transpulmonary pressure comparable to preoperative conditions. This analysis relies critically on knowledge of the patient's preoperative lung compliance (Miserocchi et al. 2010).

Furthermore, postoperative assessment of lung compliance—readily measurable intraoperatively with the chest open—has been strongly advocated as a key mechanical index to guide postoperative management and to prevent disturbances in pleuro‐pulmonary fluid balance (Salito et al. 2014, 2016).

### 5.3 Heart Failure Syndromes

The left and right ventricles are arranged in series and must therefore deliver the same cardiac output, a requirement that is far from trivial given the marked fluid‐dynamic differences and the distinct structure–function relationships of the two chambers. Arterial pressure profiles reflect peripheral resistances (afterload), which are approximately fivefold higher in the systemic than in the pulmonary circulation. Moreover, depending on functional conditions, vascular resistance may increase by ~50% in the systemic circulation but by as much as three‐ to fourfold in the pulmonary circulation.

The pulmonary circulation contains a relatively small blood volume (~450 mL), about one‐ninth of that in the systemic circulation. Assuming a heart rate of 70 beats min^−1^ and a stroke volume of 70 mL, a reduction of left‐ventricular stroke volume by only 1% would theoretically lead to a doubling of pulmonary blood volume within ~10 min. This simple estimate highlights that the autoregulatory mechanisms maintaining equality between right and left cardiac output must rely on tight proportional control based on short‐term variations in ventricular volumes. Such control mechanism is still unknown although it is known that for each ventricle the stroke volume is proportional to diastolic filling (Frank Starling law of the heart).

From a pathophysiological perspective, ventricular dysfunction is classically divided into systolic dysfunction, related to impaired contractile ability and reduced systolic pressure generation, and diastolic dysfunction, related to increased ventricular wall stiffness requiring higher filling pressures during diastole (Grossman 2000). Importantly, both systolic and diastolic dysfunction of the left ventricle are associated with edemagenic conditions in the lung.

A progressive decrease in left‐ventricular compliance reduces end‐diastolic volume and, consequently, stroke volume. The resulting backward transmission of pressure to the left atrium promotes elevation of pulmonary venous pressure, recruitment of the pulmonary capillary bed, and a potential increase in both capillary pressure and capillary surface area, thereby favoring fluid filtration across the ABB. Left heart failure is, therefore, dominated by the risk of acute pulmonary edema. In inflammatory acute lung injury, a concomitant increase in microvascular permeability represents an additional powerful edemagenic factor.

Within the framework of the $\frac{{\mathrm{DO}}_{2}}{\beta \dot{Q}}$ model, left heart failure is primarily characterized by an acute reduction in ${\mathrm{DO}}_{2}$ reflecting impaired diffusive capacity of the ABB due to interstitial and/or alveolar edema, with secondary consequences on cardiopulmonary coupling and gas exchange efficiency.

## 6 Heart–Lung Interactions in Mechanical Ventilation

### 6.1 Capillary Blood Flow

Gas exchange depends on continuous blood flow through the pulmonary capillaries, a phenomenon historically modeled as “sheet flow” (Fung 1974; Fung et al. 1983). Total blood volume in the capillary network is the range of 150–300 mL (Miserocchi et al. 2008). Pulmonary capillary occlusion, and thus interruption of *sheet flow*, occurs when alveolar pressure exceeds capillary blood pressure. The patency of pulmonary capillaries as a function of lung tissue stress and alveolar pressure has been extensively studied, particularly under zone 2 conditions (Fung 1974; Fung et al. 1983). These studies also addressed the phenomenon of endothelial cell adherence following cessation of blood flow, noting that an external force is required to separate adhered cells (Fung and Yen 1986).

Subsequent investigations demonstrated that intermittent capillary flow can induce perivascular interstitial edema, likely reflecting subatmospheric perimicrovascular pressures resulting from increased tissue stress needed to reopen the capillaries (Webb and Tierney 1974). Moreover, repeated capillary flow and pressure fluctuations have been reported to cause severe lung injury, characterized by major increases in vascular permeability and pronounced ultrastructural damage (Katira et al. 2017; Shah and Katira 2023). Recent data demonstrate cyclical “on–off” flow in pulmonary microcirculation depending on alveolar driving pressure on inspiration (Chen et al. 2015).

An additional consideration is that fluid loading, commonly employed to maintain cardiac output, will contribute to shorten capillary transit time, potentially impairing alveolar‐capillary equilibration (Figure 11B; Miserocchi et al. 2022; Shah and Katira 2023).

### 6.2 Edemagenic Factors

Overdistension of the alveolar septa is a strong edemagenic factor for lung volume exceeding 70% of Total Lung Capacity (Miserocchi et al. 2008) corresponding to a *P*_alv_ > 15 cmH_2_O (Dreyfuss et al. 1988; Miserocchi et al. 2024; Knudsen et al. 2023). Further, the increase in alveolar surface distension has been shown to increase *L*_p_ and decrease σ (Equation 2) (Parker et al. 1990; van Kaam 2024).

The inflammatory dependent increase of *P*_*γ*_ and of microvascular permeability obviously represents potent edemagenic factors as they increase transvascular flows for any given Starling gradient.

One shall recall that hyperoxia is harmful to the integrity of epithelial cells and leads to tissue matrix damage causing increased alveolar permeability (Matalon and Egan 1981; Matalon and Cesar 1985; Kolliputi et al. 2010; Ruan et al. 2020; Liang et al. 2023; Chen et al. 2024; van Kaam 2024). Further, hyperoxia leads to matrix disassembly and surfactant inactivation; no surprise that the same events occur in hypoxia, as the common cause is the production of reactive oxygen species (Miserocchi et al. 2001).

### 6.3 Impact on Gas Exchange

In acute hypoxemic respiratory failure (AHRF) patients under mechanical ventilation, a higher 30‐day mortality was correlated with low compliance, higher PaCO_2_, and higher plateau pressure (Bennett et al. 2025).

**FIGURE 16:**
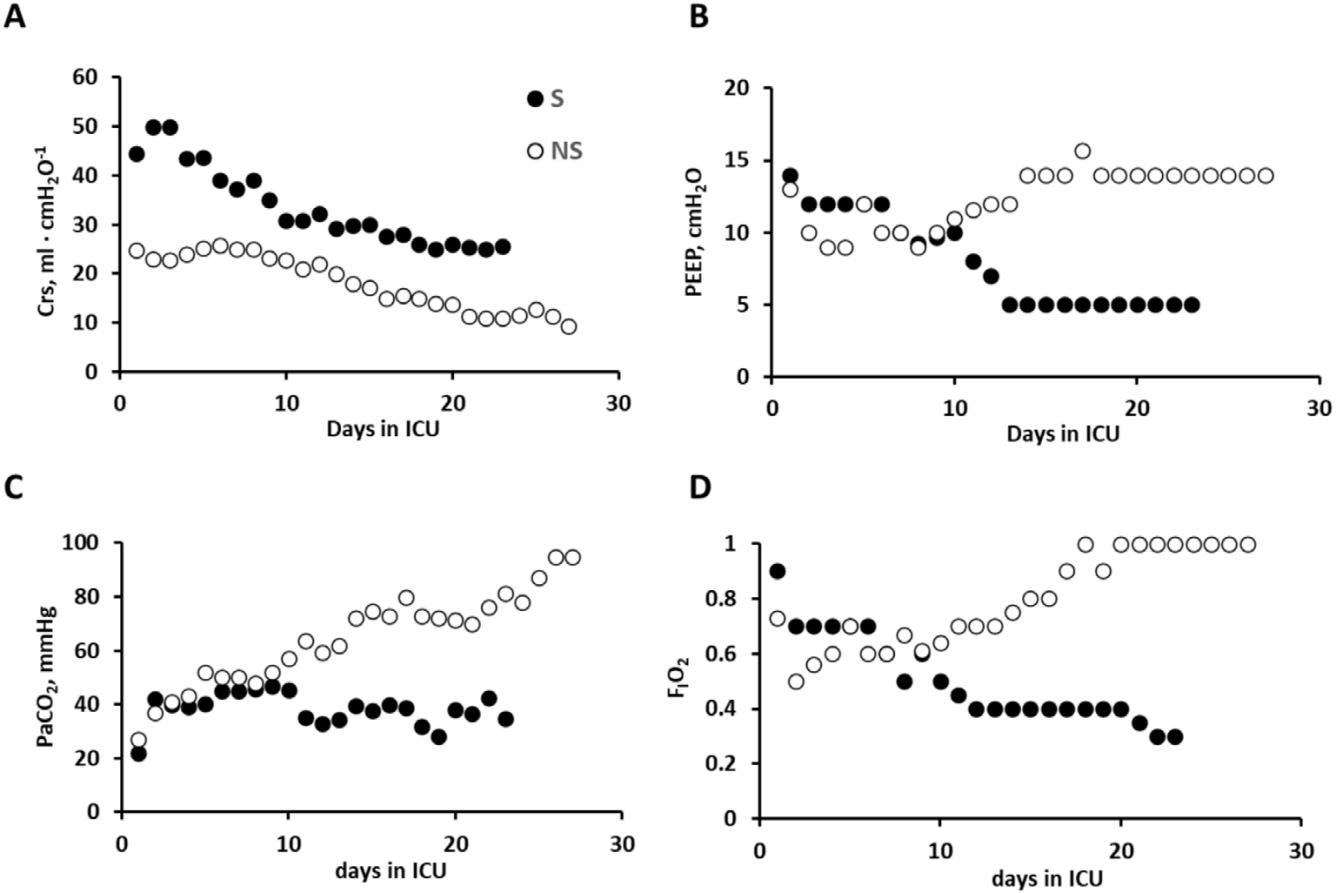
Data referring to the time course of gas exchange in the two representative patients: Survivors, S (closed symbols), and non‐survivors (NS, open symbols) in all panels: Crs (A), F_I_O_2_ (B), PaCO_2_ (C), and PEEP (D). Modified from Miserocchi et al. (2024).

The efficiency of gas exchanges in relation to the ventilatory strategy and the severity of the disease was recently estimated following the daytime evolution of respiratory parameters in mechanically ventilated COVID‐19 patients either survived (S) or not survived (NS) (Miserocchi et al. 2024). In both S and NS patients, a remarkable decrease in respiratory compliance (Crs) was observed, revealing a proportional decrease in inflatable alveolar units (Figure 16A).

All patients were hyperventilated and their SatO_2_ was maintained at > 90% by adjusting F_I_O_2_. Figure 16B shows that, over time, F_I_O_2_ was progressively decreased in S patient, while it was increased up to 1 in NS patient. It is clear that the uptake of oxygen, a gas with low diffusive and soluble properties, requiring the use of increased F_I_O_2_ is the indisputable proof of an existing O_2_ transfer limitation in mechanically ventilated patients. In these patients, the ratio $\frac{{\mathrm{DO}}_{2}}{\beta \dot{Q}}$ was decreased reflecting both the loss of alveolar units contributing to O_2_ exchange and, possibly, the development of lung edema. It is noteworthy that, in mechanical ventilation, the development of a perturbation in lung fluid balance cannot currently be diagnosed by the available clinical tools. We wish to recall that the use of FOT allowed to detect the development of even subclinical perturbations in lung fluid balance in parallel with corresponding changes in decrease in lung compliance (Dellacà et al. 2008; Bartesaghi et al. 2014).

Concerning PaCO_2_ (Figure 16C), it remained steady in S patients, while it progressively increased in NS patients. The obstacle to CO_2_ removal (a highly diffusible and soluble gas) was attributed to shortening of transit time (Figure 11B, the case of perfusion limitation). This was likely caused by increased blood velocity due to capillary squeezing induced by the increase in alveolar pressure (Figure 16D).

When Crs is remarkably decreased, high F_I_O_2_ and lung overdistension should be carefully balanced in patients under mechanical ventilation aiming to preserve the function of the gas exchanging alveoli units.

## Funding

The authors have nothing to report.

## Conflicts of Interest

The authors declare no conflicts of interest.

## Data Availability

Data sharing is not applicable to this article as no datasets were generated or analyzed during this study.
